# Cost-effectiveness of a structured progressive task-oriented circuit class training programme to enhance walking competency after stroke: The protocol of the FIT-Stroke trial

**DOI:** 10.1186/1471-2377-9-43

**Published:** 2009-08-13

**Authors:** Ingrid GL van de Port, Lotte Wevers, Hanneke Roelse, Lenneke van Kats, Eline Lindeman, Gert Kwakkel

**Affiliations:** 1 Centre of Excellence for Rehabilitation Medicine Utrecht, Rehabilitation Centre De Hoogstraat, Utrecht, The Netherlands; 2 Department of Neurology and Neurosurgery, Rudolf Magnus Institute of Neuroscience, University Medical Centre, Utrecht, The Netherlands; 3 VU University Medical Centre, Department of Rehabilitation Medicine, Amsterdam, The Netherlands

## Abstract

**Background:**

Most patients who suffer a stroke experience reduced walking competency and health-related quality of life (HRQoL). A key factor in effective stroke rehabilitation is intensive, task-specific training. Recent studies suggest that intensive, patient-tailored training can be organized as a circuit with a series of task-oriented workstations.

Primary aim of the FIT-Stroke trial is to evaluate the effects and cost-effectiveness of a structured, progressive task-oriented circuit class training (CCT) programme, compared to usual physiotherapeutic care during outpatient rehabilitation in a rehabilitation centre. The task-oriented CCT will be applied in groups of 4 to 6 patients. Outcome will be defined in terms of gait and gait-related ADLs after stroke. The trial will also investigate the generalizability of treatment effects of task-oriented CCT in terms of perceived fatigue, anxiety, depression and perceived HRQoL.

**Methods/design:**

The multicentre single-blinded randomized trial will include 220 stroke patients discharged to the community from inpatient rehabilitation, who are able to communicate and walk at least 10 m without physical, hands-on assistance. After discharge from inpatient rehabilitation, patients in the experimental group will receive task-oriented CCT two times a week for 12 weeks at the physiotherapy department of the rehabilitation centre. Control group patients will receive usual individual, face-to-face, physiotherapy. Costs will be evaluated by having each patient keep a cost diary for the first 24 weeks after randomisation. Primary outcomes are the mobility part of the Stroke Impact Scale (SIS-3.0) and the EuroQol. Secondary outcomes are the other domains of SIS-3.0, lower limb muscle strength, walking endurance, gait speed, balance, confidence not to fall, instrumental ADL, fatigue, anxiety, depression and HRQoL.

**Discussion:**

Based on assumptions about the effect of intensity of practice and specificity of treatment effects, FIT-Stroke will address two key aims. The first aim is to investigate the effects of task-oriented CCT on walking competency and HRQoL compared to usual face-to-face physiotherapy. The second aim is to reveal the cost-effectiveness of task-oriented CCT in the first 6 months post stroke. Both aims were recently recommended as priorities by the American Hearth Association and Stroke Council.

**Trial registration:**

This study is registered in the Dutch Trial Register as NTR1534.

## Background

The number of stroke patients is rising worldwide. The number in The Netherlands has been estimated at 7.5 per 1000, resulting in a prevalence of 118,500 in the year 2000[1]. Costs related to the care of stroke patients are extremely high and estimated to exceed one billion Euros in The Netherlands alone[2]. These numbers are expected to increase by 27% in 2020, due to improved health care and the ageing population[1].

Stroke patients experience lifetime disabilities and need care over a long period of time to cope with the consequences of their stroke. One study found that after rehabilitation, 62% of stroke patients were still dependent regarding daily living activities (ADL) and 32% were inactive regarding Instrumental ADLs at three years post stroke[3]. Moreover, about one fifth of chronic stroke patients suffer a significant decline in mobility[4], whereas less than 50% of patients manage to walk independently in the community again[5,6]. A qualitative study showed that loss of independent ambulation, especially outdoors, was one of the most disabling aspects for stroke patients[7]. Mobility-impaired patients are prone to inactivity and often have a sedentary lifestyle, which may result in a variety of problems including deteriorating gait, social isolation, feelings of depression and fatigue, ultimately affecting their quality of life[4]. In addition, reduced exercise tolerance as a result of inactivity may lead to secondary complications in stroke survivors, such as reduced cardio-respiratory fitness, muscle atrophy, osteoporosis and impaired circulation in the lower extremities[8]. Moreover, many patients with stroke suffer from diabetes mellitus, high blood pressure, body mass index above 30 and coronary heart disease, factors that in themselves increase the risk of other cardiovascular events, including recurrent strokes [9-11]. The lack of walking competency and physical fitness after stroke due to inactivity is of great concern to the health care system, as it is presumably associated with increasing costs[8].

A number of studies have shown that walking competency can be improved when practice is delivered in an intensive, and preferably task-oriented way [12-19]. For example, a meta-analysis of 13 RCTs (N = 501) showed that rehabilitation services in which the physical training is applied in a progressive and preferably task-oriented way are more effective than usual individual care, in terms of gait speed and walking distance[18]. Unfortunately, most stroke physiotherapists and occupational therapists are hampered in the implementation of intensive, task-oriented training by lack of time due to insufficient staff and inefficient use of human resources[20,21]. Group training, in which multiple patients practice meaningful tasks simultaneously, may be an effective method to overcome this problem. A recent meta-analysis showed that task-oriented circuit class training (CCT), in which training is organized as a circuit with a series of workstations, had positive effects in terms of gait speed, walking distance and the timed-get-up and go test, compared to usual care[19].

Task-oriented CCT generally seems to satisfy at least three key conditions for an effective and efficient physical training programme compared to usual face-to-face training. First, the use of different workstations in task-oriented CCT allows patients to practice intensively in a meaningful and progressive scheme suited to their individual needs. Second, task-oriented CCT might represent an efficient use of therapists' time while patients actively engage in task practice, compared to individual therapy. This makes circuit class training a potentially effective method which saves costs to the health care system by reducing staff-to-patient ratios. Third, task-oriented CCT involves group dynamics including peer support and social interaction[22].

Unfortunately, the literature offers only a few high-quality studies on the effectiveness of task-oriented CCT, whereas studies investigating its cost-effectiveness are still lacking[14,17,19]. The American Hearth Association recently emphasized the need for research to explore the health benefits and cost-effectiveness of structured exercise programmes to improve physical fitness in patients with stroke[8].

The study whose protocol is presented here intends to investigate the effectiveness and cost-effectiveness of task-oriented CCT programmes compared to usual face-to-face physiotherapy provided during outpatient physiotherapy treatment at a rehabilitation centre. We hypothesize that task-oriented CCT programmes will be a better strategy to improve functional outcome in patients discharged to the community from a rehabilitation centre than individual face-to-face training at the physiotherapy department of an outpatient rehabilitation clinic. In addition, based on the rules for reimbursement by insurance companies, we assume that training in groups will entail lower short-term and long-term costs to the health care system than face-to-face treatments applied individually by a physiotherapist.

## Methods/design

### Design

The effects of the task-oriented CCT programme will be investigated by means of a stratified, multicentre, single-blinded, randomized controlled trial (RCT) conducted by specially trained staff in nine selected rehabilitation centres in The Netherlands. Within each participating rehabilitation centre, patients will be allocated to the task-oriented CCT (experimental group) or regular face-to-face physiotherapy (control group) for outpatient rehabilitation.

### Setting

The study will be conducted in nine rehabilitation centres in The Netherlands ('De Hoogstraat', Utrecht; 'Heliomare', Wijk aan Zee; 'Rijnlands Revalidatiecentrum', Leiden; 'Sophia Revalidatie', Den Haag; 'Stichting Revalidatie Breda', Breda; 'Via Reva/Kastanjehof', Apeldoorn; 'Roessingh', Enschede; 'De Trappenberg', Huizen and 'Vogellanden', Zwolle). All patients will complete an inpatient rehabilitation period and will be included when they start their outpatient rehabilitation period.

### Participants

Eligible subjects will have to meet the following inclusion criteria: (1) verified stroke according to the WHO definition[23], (2) ability to walk a minimum of 10 m without physical assistance from a therapist. (i.e., patient may require verbal supervision or stand-by help from a person, and using an aid or orthotics is allowed) (Functional Ambulation Categories ≥ 3); (3) discharged home from a rehabilitation centre; (4) need to continue physiotherapy during outpatient care to improve walking competency and/or physical condition; (5) giving informed consent and being motivated to participate in 24 fitness training sessions over a 12-week period, or in usual care. Patients will be excluded if they (1) suffer from severe cognitive deficits as evaluated by the Mini-Mental State Examination (<24 points); (2) are unable to communicate (i.e. < 4 points on the Utrechts Communicatie Onderzoek) or (3) live more than 30 km from the rehabilitation centre. Before discharge from an inpatient rehabilitation setting, patients will be recruited by their own physician, and after they have given written informed consent, an observer will verify if all inclusion criteria have been met to participate in the trial. The FIT-Stroke protocol has been approved by the Medical Ethics Committee of the University Medical Centre Utrecht and all the participating rehabilitation centres. The trial is registered in the Dutch Trial Register (NTR1534).

### Definitions used in FIT-Stroke

*Task-oriented circuit class training *is defined in the present study as therapy provided to at least 2 participants simultaneously, which involves a series of workstations focusing on gait practice and functional gait-related tasks. The workstations are organized as a circuit, and the exercise at each workstation has to be progressive, i.e., increasing the number of repetitions completed at a workstation and/or increasing the complexity of the exercise performed at each station[19]. Circuit class training allows staff-to-patient ratios to be lower than they are in individual physiotherapy and enables a group of patients to exercise at different workstations simultaneously under the supervision of one or more therapists[24].

*Walking competence *is defined as **'**the ability to perform gait and gait-related tasks successfully', with gait-related activities defined as activities involving mobility-related tasks such as stair walking, turning, making transfers, walking quickly and walking specified distances'[18]. *Independent gait *is defined as level three or higher according to the Functional Ambulation Categories (FAC). FAC 3 reflects that patients require verbal supervision or stand-by-help from one person without physical contact, whereas FAC 4 indicates that patients are safe walkers on level ground, but require help on stairs, slopes or uneven surfaces, and FAC 5 means that patients are able to walk independently anywhere[25,26].

The proposed study will determine *cost-effectiveness *by relating the costs to the effects of the programme. The effects will be expressed as quality-adjusted life years (QALYs) determined by the EQ5D. The costs include the costs related to resource use of primary care practitioners, secondary care appointments, admissions to health care facilities, community-based support and individual out-of-pocket expenditure (direct costs). Medication costs and costs related to devices and adaptations in and around the house will also be included. The costs of productivity losses (indirect costs) will not be included in the analysis. The incremental cost-effectiveness ratio (ICER) will be determined by dividing the mean difference in costs by the mean difference in effect between the two groups.

### Procedure

**Figure 1:**
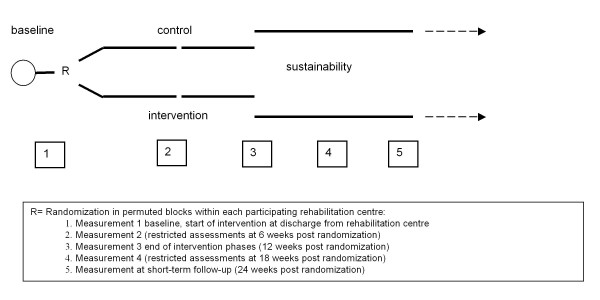
**Flow chart**.

Patients will be stratified by rehabilitation centre and subsequently randomized to task-oriented CCT or to individual face-to-face physiotherapy, using an 'online' minimization procedure[27]. The intervention period will be 12 weeks and measurements will be taken at baseline, 6, 12, 18 and 24 weeks. All outcomes measured at baseline, 12 and 24 weeks will be assessed in face-to-face meetings by an independent researcher, blinded for treatment allocation, whereas at 6 and 18 weeks, a restricted set from the test battery will be assessed by semi-structured telephone interviews (Figure 1).

### Intervention group

Patients assigned to the intervention group (two participants or more) will receive a 90-minute structured progressive task-oriented CCT programme twice a week over a twelve-week period (24 sessions). The programme includes 4 stages: (1) warming up (5 minutes), (2) circuit class training (60 minutes), (3) evaluation and a short break (10 minutes) and (4) group game (15 minutes). The training programme includes 8 different workstations, intended to improve meaningful tasks related to walking competency, such as balance control, stair walking, turning, transfers and speed walking. The eight workstations incorporated in the circuit are: (1) standing and reaching; (2) stair walking including transfer; (3) walking and picking up various objects from the ground; (4) kicking a ball; (5) stepping up and down; (6) walking course with obstacles; (7) transfers (lying to standing and sitting); and (8) speed walking. Graded progression will be achieved by (1) increasing the difficulty of the task; (2) adding weights; or (3) increasing the number of repetitions. No special (fitness) equipment is needed to perform the tasks. Each workstation will be done for 3 minutes, followed by 3 minutes of rest and 1 minute to change to the next workstation. The participants will complete the exercises in pairs, where one does the exercise while the 'partner' has a rest period and helps the other by keeping track of the number of repetitions and stimulates him/her to perform at their best. Time will be kept by the supervisors. The precise composition of the treatment package for each patient, in terms of appropriate selection of type of workstation, number of repetitions and intensity, will be determined at baseline, based on patients' profiles in terms of muscle strength, physical fitness and mobility status. All patients will keep an activity log in which they record the number of repetitions at each workstation during the sessions, which they will then use as feedback in the next session. The one-hour session of workstation training will be followed by a 15-minute group game, in which the whole group performs a game to improve walking competency. Games will vary across the sessions. Options include a game in which walking tasks are combined with a cognitive task, or a game consisting of fast walking and changing directions. Ball games can of course be used as well, as long as they serve to train walking performance. Incidents that occur during the training sessions (e.g. falls) will be reported by the supervisors to the independent investigator and registered in a falls diary. Patients who are allocated to the intervention group will receive no other physiotherapeutic treatment for the lower extremities.

### Control group

Patients who are allocated to the control group will receive regular care and will therefore be referred to the usual outpatient face-to-face treatment given by physiotherapists at the rehabilitation centres. Treatment designed to improve standing balance control, physical condition and gait performance will be provided. No additional restrictions will be imposed with respect to content, time or duration of the therapy.

### Compliance

In order to conduct this trial uniformly in nine different rehabilitation centres, all supervisors have to be trained in a one-day course before the FIT-Stroke trial starts. In the past year, about 60 physiotherapist and sports therapists have been trained. Each therapist has been informed about: (1) the criteria for recruitment of candidates discharged from the inpatient rehabilitation centre; (2) the aims, design and measurement instruments of the FIT-stroke trial, (3) how to fill in the patients' training diaries, (4) the focus and content of the task-oriented CCT programme and (5) how to stimulate the participants to maintain an active lifestyle after the task-oriented CCT sessions end. After practical training about applying and adapting the eight workstations and how to intensify the workload progressively, all trained supervisors were able to conduct the intervention in a standardized manner. Subsequently, all other physiotherapists, sports therapists and physicians who will be involved in the treatment of the included stroke patients were informed about the content of the intervention by means of presentations in each rehabilitation centre. In order to prevent contamination, therapists were instructed not to change the treatment procedures of the usual face-to-face physiotherapy during the trial. Finally, all managers of rehabilitation centres were asked not to change treatment policies during the FIT-Stroke trial. In the coming years, retraining activities will be organized and an independent investigator (IvdP) will monitor on a regular basis whether the content of the task-oriented CCT and the usual physiotherapy are being implemented as intended in the nine rehabilitation centres.

### Outcome measures

The following descriptive variables will be used for the FIT-Stroke Trial: (1) Utrechts Communicatie Onderzoek (UCO). The UCO assesses patients' ability to communicate on a five-point Likert scale, ranging from 'not able to communicate' to 'no communication problems'[28]. (2) Cumulative Illness Rating Scale (CIRS). Co-morbidity will be assessed by the CIRS, which is a valid and reliable instrument that addresses all relevant body systems without using specific diagnoses[29]. (3) Mini Mental State Examination (MMSE). Cognitive functioning will be determined by the MMSE[30]. A score lower than 24 points indicates the presence of cognitive problems[31].

The effect of the intervention will be measured at different outcome levels to determine effectiveness and cost-effectiveness. Primary outcome measure will be the mobility domain of the Stroke Impact Scale (SIS, version 3.0) and the EuroQoL.

#### Stroke Impact Scale (SIS) version 3, Mobility domain

The SIS is a self-reported, stroke-specific measure that includes 59 items and assesses 8 domains related to activities and participation[32]. The mobility domain of the SIS includes 9 questions about patients' perceived competency to keep their balance, to transfer, to walk indoors and climb stairs, to get in and out of a car and to move in their own community. Each item is scored from 'not difficult at all' to 'cannot do it at all' on a 5-point scale. A difference of 10 points on the 'mobility at home and in the community' domain of the SIS is regarded as clinically relevant[33]. SIS has shown excellent clinimetric properties in terms of concurrent and construct validity, test-retest reliability and responsiveness[33,34]. Proxies can also provide valid information on SIS outcome[34]. The SIS has recently been translated into Dutch and the Dutch version showed similar clinimetric properties when re-tested in 27 stroke patients[35]. The SIS will be applied at baseline and 6, 12, 18 and 24 weeks after randomization.

#### EuroQoL

The EuroQol is a generic measure of quality of life (QoL) and consists of a health status profile in five domains (EQ-5D), including two that are directly related to mobility[36]. It also includes a visual analogue scale (VAS) reflecting patients' overall judgement about their current health status. The EuroQol has been validated for use in stroke[37,38]. The advantage of the EuroQol is that it can be transformed into a utility score based on patients' VAS scores and population estimates of the health-status profile, allowing cost-effectiveness assessment. The VAS score will be used to detect possible overall effects of the training programme, compared to usual care, that were not specifically targeted by the intervention programme. For short-term effects, the EuroQol will be assessed at baseline and 6, 12, 18 and 24 weeks after randomization.

#### Secondary outcome measures are the following

Stroke Impact Scale (SIS) version 3.0, other domains

The other 7 domains of the SIS 3.0 will be assessed as secondary outcome measures at baseline and 6, 12, 18 and 24 weeks after randomization

#### Motricity Index (MI)

The MI is a valid and reliable measure[39] and will be used to determine the strength of the upper (MI arm) and lower paretic limb (MI leg). Scores range from 0 (no activity) to 33 (maximum muscle force) for each dimension. The test has proved highly reliable and valid[39]. The MI will be assessed at baseline and 12 and 24 weeks post randomization.

#### Functional Ambulation Categories (FAC)

Walking ability will be determined using the FAC. It includes six categories with scores ranging from 0 to 5, viz. from unable to walk to independently walking without physical assistance[26,40], though only patients with FAC 3 or higher will be recruited for the FIT-stroke trial. The test will be applied at baseline and 12 and 24 weeks post randomization.

#### Six-minute walking test

The effects of functional fitness training on gait performance and endurance will be assessed by the 6-minute walking test (6-MWT)[41]. The test will be applied at baseline and 12 and 24 weeks post randomization.

#### Five-metre timed walk

Gait speed will be measured by the 5 meter comfortable walking speed test. Gait speed is responsive to change and closely related to walking performance in hemiplegic patients[40]. In order to reduce measurement error, the mean of three repeated walking speed measurements will be calculated[25]. Patients will rest for about one minute between each test. Using a digital stopwatch that records time within 0.01 second, timing will be manually started at the 'go' instruction and stopped when the subject crosses the 5 meter mark. The test will be applied at baseline and 12 and 24 weeks post randomization.

#### Timed balance test (TBT)

The TBT consists of 5 different components on an ordinal scale and involves timed balance (i.e., 60 seconds) on progressively diminishing support surfaces. The test has been shown to be reliable and closely related to walking performance[40,42]. The test will be used at baseline and 12 and 24 weeks post randomization.

#### Timed up and go test (TUG)

The TUG is a test of basic functional mobility. The participant is asked to rise from an armchair, walk 3 m as fast as possible, cross a line, turn, walk back and sit down again. The time taken to perform this task is recorded. The TUG has been shown to identify patients at increased risk of falls[43]. The test will be applied at baseline and 12 and 24 weeks post randomization.

#### Modified stairs test

The modified stairs test is an extended version of the Timed up and go test (TUG). The test includes the same tasks as the TUG, plus ascending and descending 5 steps[44]. Patients are timed to the nearest 0.01 s from the moment they are asked to rise from a chair which is placed 0.5 meters in front of the stairs, ascend 5 steps, turn and descend the 5 steps and sit down again in the chair as safely as possible. Patients rest for about one minute between each test. The test will be applied at baseline and 12 and 24 weeks post randomization.

#### Rivermead Mobility Index (RMI)

The RMI consists of 14 questions and one observation, covering aspects ranging from turning in bed to running[45]. Questions are simple and are scored dichotomously. The measure is reliable, valid and responsive [45-47]. The RMI will be applied at baseline, 12 and 24 weeks post randomization.

#### Nottingham Extended ADL (NEADL)

The NEADL scale[48] is based on a self-reported questionnaire on levels of activity actually performed. The NEADL consists of 22 items in 4 domains (mobility, kitchen, domestic, leisure). The NEADL is specifically designed for postal use with stroke patients, and has proved to be reliable and valid as an outcome measure in trials and observational studies. Each item is rated by one of four responses (able, able with difficulty, able with help, unable). The scale has been shown to have reasonable hierarchical (ordinal) properties in stroke patients. The NEADL will be assessed at baseline and 12 and 24 weeks post randomization.

All of the above tests, for both the experimental and control groups, will be implemented at the participants' own home by an independent observer who is blinded to treatment allocation.

#### Falls Efficacy Scale (FES)

The FES is an instrument to measure fear of falling, based on the operational definition of this fear as 'low perceived self-efficacy at avoiding falls during essential, nonhazardous activities of daily living'[49]. The FES will be assessed at baseline and 12 and 24 weeks post randomization.

#### Hospital Anxiety and Depression Scale (HADS)

The HADS is a simple measure to determine mood, emotional distress, anxiety, depression and emotional disorder. It is a brief, valid, reliable and widely used measure, known to produce meaningful results as a psychological screening tool. The HADS consists of 14 items (7 anxiety, 7 depression), each with a 4-point rating scale (0–3) and has proved to be responsive to change[50,51]. The HADS will be assessed at baseline and 12 and 24 weeks after randomization.

#### Fatigue Severity Scale (FSS)

The FSS will be used to assess the impact of fatigue. The FSS consists of 9 items, and scores for each item range from 1 to 7. The total FSS score is the mean of the 9 item scores[52]. In a reliability study with two independent observers and 18 stroke patients, FSS showed an intra-class correlation coefficient (ICC) of 0.82[53]. The FSS will be applied at baseline and 12 and 24 weeks post randomization.

#### Letter cancellation task

Inattention will be measured by the letter cancellation task[54] and will be scored positive when patients score three omissions or more on one side, compared to the other side.

#### Diaries

Finally, each patient will be asked to keep a cost diary, which will be used to assess medical consumption in both arms of the study. Patients will be asked to record their medical consumption (for example visits to a general practitioner, hospital visits, medication intake) each week. In addition, they will be asked to record their level of activities during the day (activity log) as well as special events (e.g. falls). This dairy will be kept daily for 12 weeks. After 12 weeks, the patients will be asked to continue recording weekly until 24 weeks after inclusion.

### Power analysis

A difference of 5 points (about 11%) on the 'mobility at home and in the community' domain of the SIS in favour of the experimental group will be regarded as clinically relevant[33], whereas the standard deviation for this population is estimated at a maximum of 14 points (28%) based on 287 mild and moderate SIS responses[55]. In addition, test-retest reliability (ICC) is known to be about 0.8[33], whereas the FIT-Stroke trial uses 4 repeated measurements in the first 24 weeks after randomization. Therefore, a minimum of 99 patients will be required for each arm of the trial. Expecting a dropout rate of 10%, we assume that a minimum of 220 stroke patients will be needed to achieve a sufficient statistical power of 80%.

### Data analysis

The present trial, with repeated measurements nested in each patient, will apply a random coefficients model (MLWIN, version 2.11) to evaluate differences in effect between the experimental and control intervention arms. Besides the type of intervention, the model will include significant factors such as covariates, which will be established by means of (univariate) regression analysis. It should be noted that Random Coefficient Analysis (RCA) is able to deal with (partially) or completely missing values and enables an 'intention-to-treat' analysis. Economic evaluation will be conducted from the main assumption underlying the present study, that the proposed fitness training programme, which allows stroke patients to train in groups of 8 to 10, will result in a better walking competency as well as improved (I)ADL and HRQoL, with net savings on resources (i.e., costs), compared to the control intervention. The balance between costs and effects will be estimated, in accordance with the Dutch guidelines for pharmaco-economic research, by means of multivariable probabilistic data analysis [56]. The cost diary will comprise detailed questions on items such as consultations with neurologists, family doctors, paramedics, home care and non-professional support, and the answers will be used in the costs evaluation. Provision of medication by pharmacies in the community will also be recorded to keep track of expenses related to drug use. The diary will also record low volumes of high-cost resources, such as re-admission to hospitals and rehabilitation centres. Ultimately, multiplication of resource use and unit costs, which will be determined in a separate cost study, will yield an estimate of patient-level cost. Subsequently, summation across individual patient records and averaging will yield an estimate of total costs per intervention arm. In addition, the impact of task-oriented CCT in terms of health-related quality of life will be determined using the EuroQol 5D (EQ-5D). The EQ-5D is a 5-item questionnaire that can be converted into a single value score (i.e., utility) for HRQoL, yielding an estimate of the quality-adjusted survival time (QALYs). This estimate will serve as the primary measure of effectiveness for the economic evaluation.

## Discussion

The FIT-Stroke trial is a single-blinded multicentre trial in which 220 patients will be allocated to task-oriented CCT or usual face-to-face treatment at the nine participating rehabilitation centres, on the basis of a minimization procedure. The minimization procedure used for patient allocation has been recommended as a highly effective allocation method for randomized controlled trials[27].

The main characteristics of the task-oriented CCT programme are the progressive intensity and task specificity of the workstations used. The eight different functional workstations used in the trial have been found to be meaningful and relevant to patients' needs, goal-oriented, challenging, and feasible (i.e., not to easy and not to difficult). As such, the programme builds heavily on the existing evidence that intensity of practice[12,57] as well as task specificity[13,57] are the main drivers of gait improvement. After reviewing 14 trials, French and colleagues[13] showed that repetitive task-oriented training resulted in modest improvement in lower limb function. Although the authors claimed that the repetitive lower limb training was sufficient to influence daily living functions, no evidence was found that the effects of repetitive training were sustained once training had ended. In order to fill this gap in existing evidence, the FIT-Stroke trial will have a follow-up of three months to investigate the possible wash-out effects of the task-oriented CCT programme.

Recently, Wevers and colleagues showed significant effects in favour of task-oriented CCT for walking distance, gait speed and a timed up-and-go test in their systematic review of randomized controlled trials including 307 participants[19]. The intensity of task-oriented CCT ranged from 4 weeks (3 times a week for 60 minutes)[24] to 19 weeks (3 times a week for 60 minutes)[58]. Therefore, we assume that in the present study, a training schedule of 24 sessions each lasting 90 minutes within 12 weeks will be sufficient to achieve functional gains in gait and gait-related activities. Unfortunately, most studies included in the above meta-analysis were small and statistically underpowered, ranging from 12[24] to 91[59] participants, which suggests that larger trials are warranted. Only one study[60] included in the meta-analysis investigated the effects of task-oriented CCT within the first 6 months post stroke. Interestingly, this study found a larger effect size than those that investigated effects in chronic stroke. However, none of the included studies compared the cost-effectiveness of task-oriented CCT with that of usual care, which is particularly important in view of the burden of health care costs for stroke and attempts to save costs in stroke management. This aim is in line with the views of the American Hearth Association (AHA), which recently recommended that fundraisers and researchers should conduct cost-effectiveness studies in the growing and ageing stroke population[8]. By including 220 patients within the first months after their stroke and focusing on cost-effectiveness as well as health-related quality of life, we hope to fill the existing gaps in stroke rehabilitation research.

Finally, we will give special attention to the generalizability of the effects of this physical intervention to psycho-social outcomes, like fatigue, anxiety and depressive symptoms. Including a large population will enable us to conduct sub-analyses on smaller groups for these less frequent outcomes. The larger population will also enable us to record the effects on other less frequent outcomes like recurrent stroke, and co-morbidities like cardiac problems. Ongoing participation in physical activity may also reduce the prevalence of vascular risk factors and so reduce the risk of coronary heart diseases and recurrent strokes[10,11]. For example, a meta-analysis[61] has demonstrated that increased physical activity improves cardiac performance and exercise capacity in patients with heart failure.

In conclusion, task-orientated CCT holds great potential for the rehabilitation of people after stroke, allowing the training schedule to be customized to the individual status of each participant. We hypothesize that task-oriented CCT will be equally effective in improving gait and gait-related activities in stroke as face-to-face therapy alone, or even more so. The FIT- Stroke trial will give us a unique opportunity to study the effects of task-oriented circuit class training in a large and carefully designed trial. The first results of the study are expected in August 2011.

## Competing interests

The authors declare that they have no competing interests.

## Authors' contributions

GK and IvdP developed the idea and procured funding for the study. All authors contributed to the design and the protocol of the study. All authors reviewed the manuscript and approved the final version.

## Pre-publication history

The pre-publication history for this paper can be accessed here:


